# Understanding Real-Time Fluorescence Signals from Bacteria and Wound Tissues Observed with the MolecuLight i:X^TM^

**DOI:** 10.3390/diagnostics9010022

**Published:** 2019-02-26

**Authors:** Monique Y. Rennie, Danielle Dunham, Liis Lindvere-Teene, Rose Raizman, Rosemary Hill, Ron Linden

**Affiliations:** 1 MolecuLight, Inc, Toronto, ON M5G 1T6, Canada; ddunham@moleculight.com (D.D.); lteene@moleculight.com (L.L.-T.); 2 Department of Professional Practice, Scarborough and Rouge Hospital, Toronto, ON M1E 4B9, Canada; rraizman@shn.ca; 3 Department of Ambulatory Care, Lions Gate Hospital, Vancouver Coastal Health, North Vancouver, BC V7L 2L7, Canada; rosemary.hill@vch.ca; 4 Judy Dan Research and Treatment Centre, North York, ON M2R 1N5, Canada; drlinden@ontariowoundcare.com

**Keywords:** bacteria, wounds, wound assessment, fluorescence imaging, image interpretation, MolecuLight i:X

## Abstract

The persistent presence of pathogenic bacteria is one of the main obstacles to wound healing. Detection of wound bacteria relies on sampling methods, which delay confirmation by several days. However, a novel handheld fluorescence imaging device has recently enabled real-time detection of bacteria in wounds based on their intrinsic fluorescence characteristics, which differ from those of background tissues. This device illuminates the wound with violet (405 nm) light, causing tissues and bacteria to produce endogenous, characteristic fluorescence signals that are filtered and displayed on the device screen in real-time. The resulting images allow for rapid assessment and documentation of the presence, location, and extent of fluorescent bacteria at moderate-to-heavy loads. This information has been shown to assist in wound assessment and guide patient-specific treatment plans. However, proper image interpretation is essential to assessing this information. To properly identify regions of bacterial fluorescence, users must understand: (1) Fluorescence signals from tissues (e.g., wound tissues, tendon, bone) and fluids (e.g., blood, pus); (2) fluorescence signals from bacteria (red or cyan); (3) the rationale for varying hues of both tissue and bacterial fluorescence; (4) image artifacts that can occur; and (5) some potentially confounding signals from non-biological materials (e.g., fluorescent cleansing solutions). Therefore, this tutorial provides clinicians with a rationale for identifying common wound fluorescence characteristics. Clinical examples are intended to help clinicians with image interpretation—with a focus on image artifacts and potential confounders of image interpretation—and suggestions of how to overcome such challenges when imaging wounds in clinical practice.

## 1. Introduction

Bacteria is a clinical challenge in acute and chronic wounds [1] which can delay or entirely prevent wound healing [2,3,4,5]. The presence of significant bacterial loads typically requires a bacterial-targeted treatment plan such as debridement or use of antimicrobials to optimize the wound bed to support healing [2,6,7]. During wound assessment, tissue types associated with different stages of a wound (e.g., necrotic tissue, slough, granulation tissue) are visible to the naked eye. Their presence provides the clinician with valuable information about a wound’s current status and its healing potential [8], which in turn guides treatment selection. In contrast, bacteria in a wound is not visible. Diagnostic confirmation of bacteria is obtained via wound sampling techniques, but this delays confirmation by several days [9], is prone to erroneous results [10,11], and is costly; for these reasons, many clinicians opt not to sample the majority of wounds [9]. At the point of care, bacterial presence at high loads is inferred based on clinical signs and symptoms [12,13,14,15]. However, these indicate a host response to bacterial loads and do not give any information on the location(s) of high bacterial loads within and around the wound. Of even greater concern is that high levels of bacteria in wounds, and even infections, have been shown by numerous clinical studies and meta-analysis to be asymptomatic in the majority of clinical cases [12,14,16].

Fluorescence imaging has recently emerged as a point-of-care and non-invasive imaging method for visualizing both wound tissue and bacterial fluorescence [17,18,19,20,21,22,23] (MolecuLight i:X, MolecuLight, Canada). By visualizing bacterial fluorescence signals in real-time, images can reveal not just the presence of bacteria but the spatial pattern of bacterial burden. This essentially creates a map of bacteria in the wound that could be used by the clinician for targeted sampling, cleaning, debridement, and other wound therapies [18,19,21,23,24].

Understanding image interpretation is important for any new imaging technology, including fluorescence. This paper is meant to identify key observations made to date in the clinical use of this novel wound imaging technology so that future clinicians can learn how to recognize key features on fluorescence images and improve their clinical interpretations of these images in practice. The clinician authors of this paper were some of the earliest adopters of this technology and have each imaged thousands of wounds with this device. Herein they share clinical examples, their experiences surrounding the use of this device to image wounds, and information on the interpretation of MolecuLight i:X fluorescence images.

## 2. How Does the MolecuLight i:X Work?

**Figure 1:**
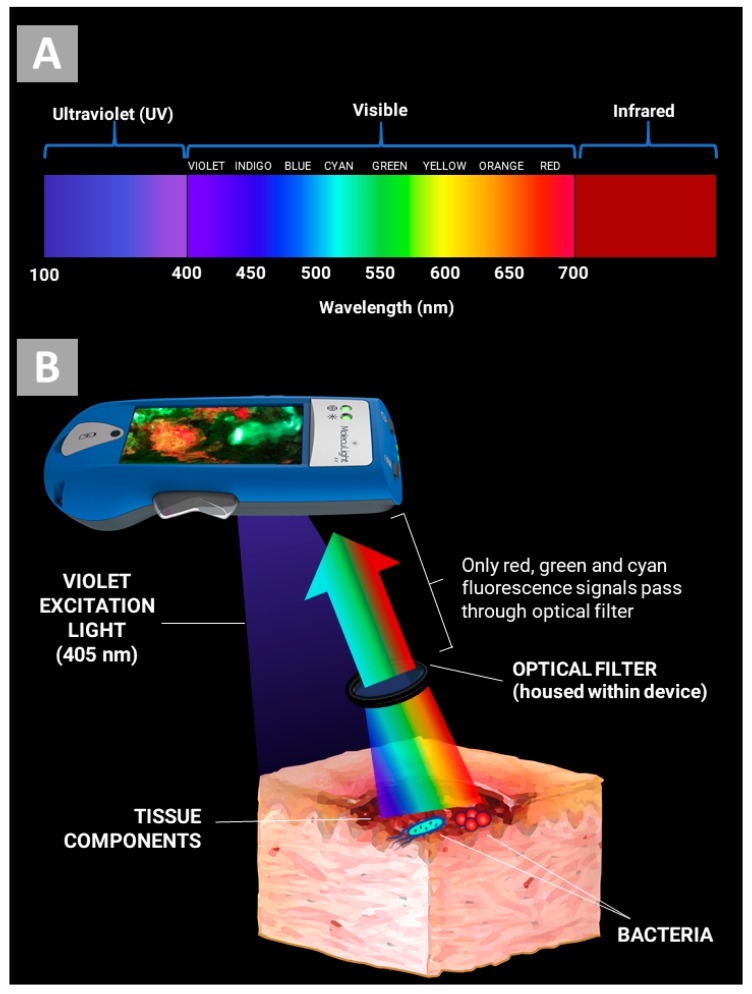
Real-time optical filtering of non-informative wavelengths of light. (**A**) Excitation of tissues and bacteria with 405 nm violet light results in a spectrum of fluorescence signals which span the spectrum of visible light. (**B**) Optical filters allow fluorescence signals only from information-rich wavelength bands and prevent reflected violet light from contaminating the image, without any digital processing.

The illumination of tissues with an excitation light produces a spectrum of fluorescence signals [25]. This is innate autofluorescence; no contrast agents are required to yield this fluorescence. The spectrum of signals produced depends on both the excitation wavelength(s) and the composition of biological and non-biological sources being excited and imaged [25,26]. It has long been known that tissue excitation with ultraviolet and violet light produces fluorescence signals with a range of colours spanning the visible spectrum (Figure 1A). In the context of wound care, some of these wavelengths/colours are tremendously informative. For example, ultraviolet and violet excitation trigger the emission of red fluorescence from porphyrins [27], which is indicative of bacteria [21]. However, intrinsic fluorophores present in the surrounding tissue also emit fluorescence signals when excited, many of which are less informative and cloud one’s ability to discern the wavelengths of interest. The wavelengths of interest could also be dampened by reflected violet light shining back at the device. For these reasons, the MolecuLight i:X device combines well-known principles of violet light excitation with advancements in optical filtration, as follows:

Biological components of wounds, including wound tissues and bacteria, are excited by the MolecuLight i:X via its built-in light-emitting diodes (LEDs), which emit a narrow band of safe 405 nm violet-colored excitation light [18,21]. Inherent bacterial porphyrins are optimally excited at 405 nm [28]. Note that, unlike ultraviolet imaging systems which are phototoxic to tissues [29], the intensity of the device’s violet light is entirely safe for clinical use [21].

Custom device optics filter out the non-informative wavelength bands of the fluorescence spectrum. Only signals from wavelengths associated with bacterial fluorescence (red and cyan) and from a narrow range of green tissue autofluorescence (for anatomical context) can pass through the optical filters to form the real-time image. Autofluorescence wavelengths emitted by 405 nm-excited tissue components span a broad range of colours in the visible spectrum, typically between 420–700 nm (including violet, blue, green, and yellow; see Figure 1A). Some examples of these autofluorescence signals are blue fluorescence from reduced nicotinamide adenine dinucleotide (NADH) and green fluorescence from flavin adenine dinucleotide (FAD). The optical filter allows only fluorescence with wavelengths between 501–542.5 nm (±1.5 nm)—wavelengths of cyan and green—and 601–664 nm (±1.5 nm)—wavelengths of red and orange—to pass through to the sensor. This is depicted in Figure 1B.

The custom fluorescence optical emission filter prevents image contamination from the reflected, high intensity excitation light. The relative intensity of the porphyrin fluorescence signal is orders of magnitude lower than the high power 405 nm violet excitation light [30]. Therefore, the absence of an optical filter to eliminate the reflected light from the image would substantially diminish the sensitivity of detecting the lower intensity bacterial fluorescence signals.

The device incorporates a built-in imaging sensor to enable digital image and video recording of a standard photo and/or fluorescence image from a wound. Note that the emission filter is placed in front of the device’s built in imaging sensor, allowing real-time capture of tissue fluorescence without digital processing of any kind.

## 3. Evidence for Bacterial Detection

**Figure 2:**
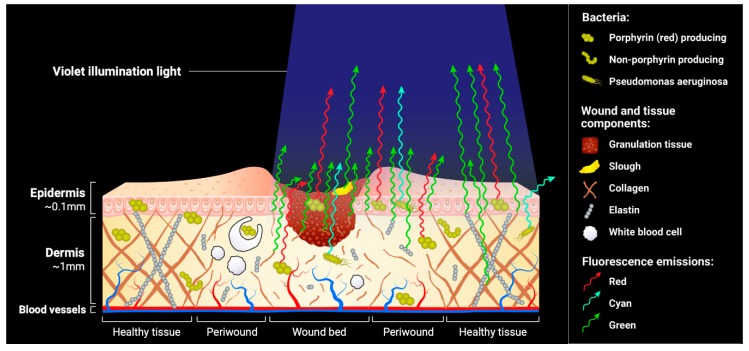
Fluorescence emissions from tissues and bacteria. When illuminated by the 405 nm violet light, extracellular matrix components emit green fluorescence, porphyrin-producing bacteria emit red fluorescence, and *Pseudomonas aeruginosa* emits cyan fluorescence. Subsurface fluorescence can be detected; however, the fluorescence emissions are blunted by optical scattering as a result of passing through overlying tissues. This is depicted by shorter fluorescence emission arrows coming from subsurface tissues and subsurface bacteria. Note that components deeper in the dermal later (>1.5 mm) will not emit detectable fluorescence signals because the violet excitation light cannot penetrate the tissue far enough to reach them.

The largest application of this fluorescence imaging device to date is the visualization of bacteria within and around wounds. Endogenous bacterial fluorescence is well-established in the literature [31,32,33]. The MolecuLight i:X device exploits the high levels of endogenous fluorophores bacterial species are known to produce—porphyrins [27,34,35,36] and pyoverdines [37]. Porphyrins are a naturally occurring, red-fluorescing by-product of bacterial haem production [27,36] and are produced by the vast majority of bacterial pathogens [38]. In contrast, cyan-fluorescing pyoverdines are fluorophores specific to the pseudomonads [39], most notably *Pseudomonas aeruginosa*, a common wound pathogen requiring early identification and unique, specifically targeted treatments [40]. Pyoverdines are produced endogenously as part of the pseudomonad iron acquisition process [37]. Both the endogenous porphyrins and pyoverdines are excited when illuminated by the device’s violet light and emit specific wavelengths of light in the red and cyan bands, respectively (Figure 2). Bacteria produce other endogenous fluorophores (e.g., from NADH and FAD) which are not depicted in Figure 2, some of which are similar to that of tissue and the majority of which do not pass through the optical filters. Based on clinical evidence to date, any non-red or non-cyan fluorescent signals from bacteria have no confounding effects on image interpretation.

Clinical studies have consistently shown that red and cyan signals visualized on the device are predictive of bacteria at moderate to heavy loads [19,21,41,42,43]. These are bacterial loads of clinical concern which are known to delay healing [2] and prohibit application of advanced therapies (e.g., grafts [44]). Multi-center clinical trials have established that the positive predictive values of red and cyan fluorescence signals observed with this device are 100% for detecting bacteria, i.e., no false positives were detected [21,42,43]. High sensitivities of red and cyan fluorescence for determining the presence or absence of bacteria have also been clinically reported in chronic wounds (e.g., diabetic foot ulcers (DFU), venous leg ulcers (VLU), pressure ulcers (PU)), burn wounds, and surgical wounds) [20,41,42,45]. These clinical results are further supported by pre-clinical studies on bacteria-inoculated mouse wounds, which unequivocally demonstrate the specificity of red and cyan signals to bacteria [22,46].

## 4. Image Interpretation

It is important that clinicians using the device are able to interpret the image meaningfully to aid in their assessment of wounds, i.e., understand the different fluorescence colours appearing on the display screen. Herein, we describe the various fluorescence colours detected from wound tissues and bacteria with this device. Note that fluorescence images should always be compared to standard images during image interpretation to provide clear anatomical context for some of the fluorescence signals being observed. Further note that the device is meant to be used adjunctively to the current standard of care; it does not replace the need for clinical assessment for signs and symptoms of infection and should not be used to rule out the presence of tunneling or other bacteria located deep under the wound.

### 4.1. Tissue Fluorescence

Tissue fluorescence in MolecuLight i:X images provides anatomical context for comparison with potential regions of bacteria as well as insight into the tissue types being imaged. Tissue autofluorescence is mainly due to endogenous fluorophores, which are fluorescent components native to tissues. There are numerous sources of fluorophores, but the predominant fluorophores in tissue are from extracellular matrix proteins (e.g., collagen, elastin, fibrin) and red blood cells. These tissue components generally fluoresce in the green and yellow portions of the visible spectrum [25,47,48]. However, when imaged with the MolecuLight i:X, tissue predominantly appears green in colour due to optical filtering of other extraneous regions of the tissue autofluorescence spectrum (e.g., yellow, blue).

**Figure 3:**
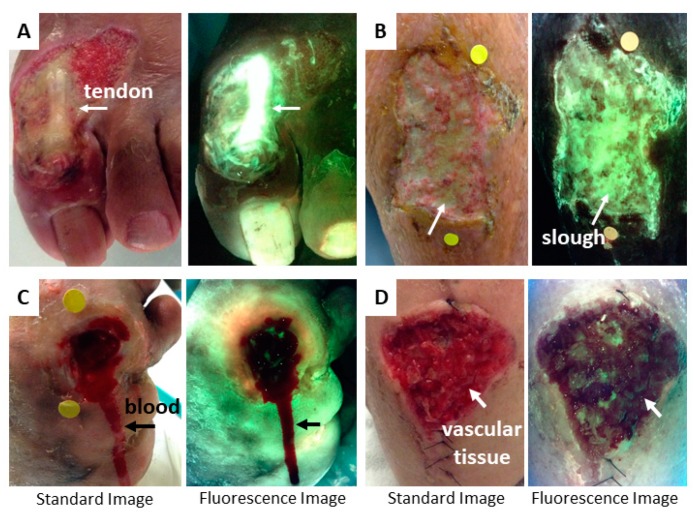
Characteristic fluorescence signals from wound tissues. (**A**) Intense green/glowing white fluorescence from a tendon due to high collagen content. This signal could be confused for *P. aeruginosa* if the clinician did not compare the fluorescence observed with the standard image of the wound. The standard image makes it obvious that the glowing white region of fluorescence is a tendon. Bright green is also shown in a toenail. (**B**) Slough, which appears yellow on a standard image, fluoresces light green in this venous leg ulcer (VLU) due to high fibrin content. (**C**) Blood from a diabetic foot ulcer (DFU) (post-debridement) appears maroon on fluorescence images due to absorption of the excitation light by hemoglobin. Blood should always be removed from the wound bed prior to imaging. Blush red fluorescence can be seen in the periwound tissue of this DFU, suggesting the presence of bacteria at moderate to heavy loads. (**D**) Highly vascular tissue (e.g., granulation tissue) also fluoresces maroon due to absorption of illumination light by hemoglobin. Note that circular yellow stickers in wounds **B** and **C** are for the device’s wound measurement software.

**Figure 4:**
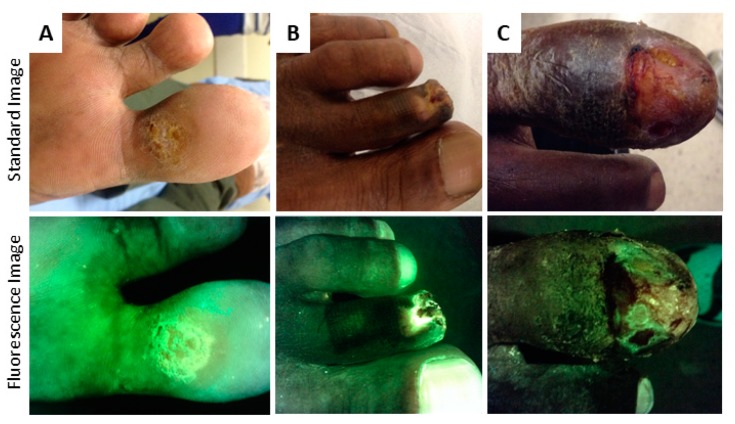
Variation in green fluorescence due to skin tone. (**A**) Lighter skin tones fluoresce a light green colour. As melanin concentration increases (**B**,**C**), the tone of green in the fluorescence images also darkens due to absorption of excitation light preferentially by melanin causing attenuation of the tissue fluorescence.

The shade of green observed will vary with tissue type. This is due, at least in part, to differences in the relative densities of collagens and elastins and their degree of cross-linking, both of which shift the peaks in a tissue’s fluorescence spectrum [25,49]. The densest, collagen rich tissue structures, tendon and bone, fluoresce a very bright and high intensity green to glowing white (Figure 3A). To confirm that this intense green or glowing white is anatomical in nature, rather than bacterial, the clinician need only to compare the pattern of fluorescence to a standard image of the same region. Slough tissue also appears bright green due to its high fibrin content [50] (Figure 3B) and can also be confirmed by comparing to the standard image in which slough appears yellow or white. Skin is a less intense green, but the specific shade varies with skin colour; darker skin tones appear darker green due to the increased absorption of some of the violet light by melanin [51] (examples in Figure 4). Flaky skin often appears green with thin white edges outlining the flakes. At the opposite end of the optical spectrum is dead or necrotic tissue lacking blood supply, in which the extracellular matrix components (collagen, elastin, etc.) have largely been decomposed. This tissue appears black both to the naked eye and on fluorescence images. Hemoglobin (Hb) in blood is a biological molecule that preferentially absorbs the 405 nm excitation light [52]. The resulting appearance on real-time fluorescence images is dark black/maroon in regions of blood (Figure 3C). Hemoglobin absorption of light between 400–700 nm of the visible spectrum can occur in highly vascular tissues (e.g., granulation tissue, Figure 3D) or from surface contamination by blood and may attenuate (i.e., reduce the intensity of) the fluorescence from bacteria. Therefore, it is important to clean any blood from the wound prior to taking a fluorescence image.

### 4.2. Bacterial Fluorescence

As described above, the device detects the high levels of endogenous fluorophores that bacterial species are known to produce, porphyrins [27,34,35,36] (red) and pyoverdines [32,37] (cyan). Porphyrins are produced by the vast majority of bacterial pathogens [38] while pyoverdines are fluorophores specific to *Pseudomonas aeruginosa* [39].

**Figure 5:**
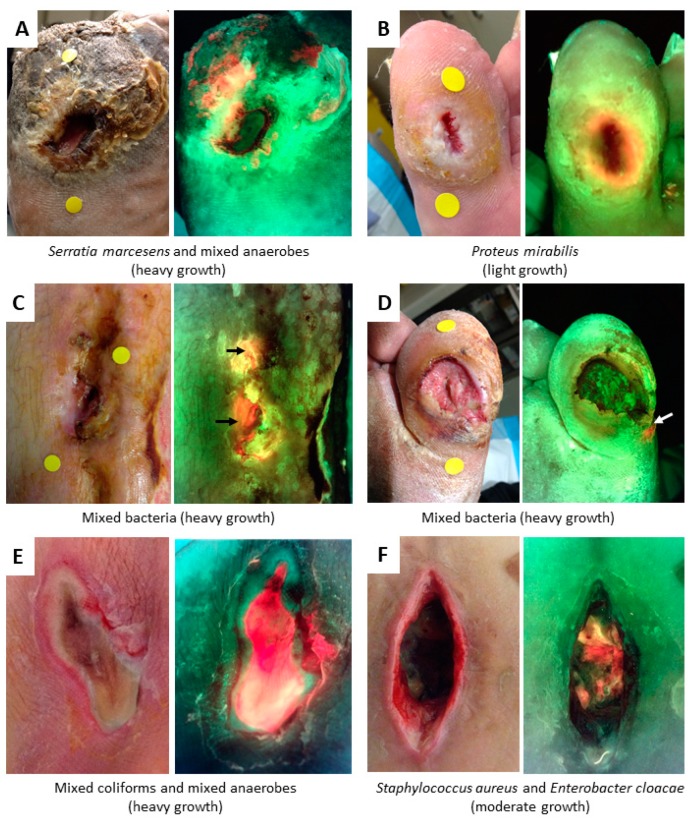
Fluorescence from bacteria appears red or pink/blush. Red fluorescence is a result of endogenous porphyrins emitted by most bacterial species when excited by 405 nm violet light. (**A**–**F**) All wounds show a standard image on left and fluorescence image on right, with microbiology results from cultures reported below. Circular yellow stickers seen in wounds **A**–**D** (standard images) are for the device’s wound measurement software. Wounds **A**, **B,** and **D** = DFU; wounds **C** and **F** = surgical; wound **E** = pressure ulcer (PU).

The colour of red fluorescence visualized is dependent on the depth of bacteria within the wound and the other tissues present in the region which contribute fluorescence to the composite image. Imaging depth is limited by the penetration of the illumination light, to a maximum of ~1.5 mm beneath the surface [18,53], though this will vary based on the tissue type being imaged [54]. Generally, the brightest red colours are from fluorescent bacteria at or close to the surface, while pink and “blush” red colours are a result of subsurface fluorescent bacteria. This is due to optical scattering of the red fluorophores as they pass through the tissues above them, as depicted schematically in Figure 2. In practice, this means that heavy bacterial loads may appear a faint blush red colour if they are located immediately subsurface; the DFU in Figure 5D is an example of this principle. The colour of red fluorescence observed may also be shifted due to the blending of fluorescence colours with the overlying tissues fluorescing bright green. For example, a bright green fluorescing callus region with red fluorescing bacteria underneath can create a region of yellow colour on the fluorescence image that clinicians may wish to inspect more carefully. Example wounds exhibiting red bacterial fluorescence, confirmed via microbiological cultures, are shown in Figure 5.

**Figure 6:**
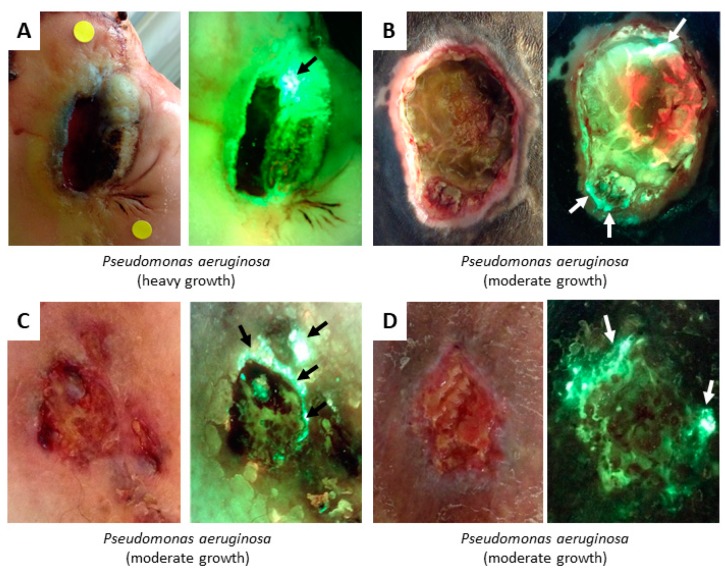
Cyan and glowing white fluorescence from *P. aeruginosa.* Endogenous pyoverdines found in *P. aeruginosa* emit cyan fluorescence when excited by 405 nm violet light. (**A**–**D**) All wounds show a standard image on left and fluorescence image on right, with arrows denoting regions of cyan fluorescence. The cyan signal can oversaturate the camera, resulting in a glowing white region, as seen in wounds **A** (black arrow), **B** (top arrow), and **C**. Wounds were sampled in regions of cyan or white fluorescence using a swab; microbiological cultures confirmed the presence of *P. aeruginosa* in all cases. Wound **A**—DFU; Wound **B**—Pressure ulcer (also demonstrates red fluorescence, but the region of red was not sampled); Wounds **C** and **D**—VLUs.

Cyan fluorescence is a vibrant blue/green colour, distinct from the duller green fluorescence of skin. It often appears in conjunction with glowing white “hot spots” (e.g., Figure 6). These are due to the cyan colour oversaturating the camera. Note that other components may cause white to appear on the fluorescence image, such as the tendon in Figure 3A or flaky skin which can present with white edges on the flakes. However, glowing white from *P. aeruginosa* is distinct due to its: (1) Blurry edges (as opposed to sharp edges on flaky skin), (2) its lack of correlation to any tissue architecture or non-biological fluorescent source on the standard image, and (3) its co-appearance with cyan colour. The representative examples of cyan and glowing white in Figure 6 were all confirmed to be from *P. aeruginosa* at moderate to heavy growth. Note that none of these four wounds exhibited the so-called “hallmarks” of *P. aeruginosa* for clinical detection (greenish tinge on the bandage, sweet smell). This suggests that fluorescence images are capturing cyan-fluorescing, but otherwise asymptomatic, *P. aeruginosa* cases which would not have been clinically detected without wound sampling.

## 5. Methods to Minimize Imaging Artifacts and Misinterpretation

**Figure 7:**
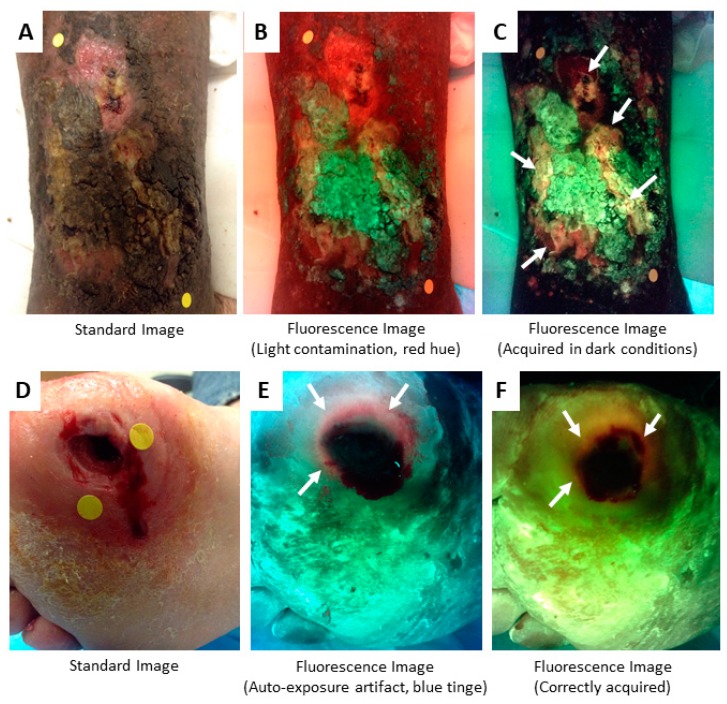
Imaging artifacts to be avoided. (**A**–**C**) Light contamination gives the fluorescence image in panel **B** a reddish tinge. This obscures detection of bacterial fluorescence, which was clearly present (arrows) when wound was imaged under dark conditions (**C**). (**D**–**F**) The violet illumination light should only be turned on after darkness has been obtained to avoid a bluish artifact from auto-exposure as seen in (**E**). When correctly acquired, as in (**F**), the skin will have a greenish hue. White arrows denote regions of red fluorescence.

All fluorescence imaging technologies are susceptible to artifacts from ambient light [55], including the MolecuLight device. Contamination with ambient light can contribute to the fluorescence captured in the red channel (600–665 nm) and lead to false positives or misinterpretation of bacterial fluorescence. The device therefore requires a dark environment for fluorescence imaging. An ambient light sensor on the device turns green when sufficient darkness for fluorescence imaging is present. Should a dark environment not be possible (e.g., due to windows in the room or other unremovable light sources), the device has an attachable drape to generate complete darkness across the imaging field. When fluorescence images have light contamination, their fluorescence colours are much more challenging to interpret, and a reddish hue often spans the entire light-contaminated region (e.g., Figure 7B). A reddish hue can also occur on standard images when the device’s optical emission filter is in place but the violet illumination light is not on; this occurs only when the illumination light auto-shuts off after 90 seconds of continuous use and can be righted by flipping the device’s illumination switch on and off. Ensure that the device’s rocker switch is in standard mode to avoid this artifact.

It is important that the violet illumination light only be turned on after darkness has been obtained in the environment, and confirmed with the ambient light sensor, to avoid a bluish artifact in the fluorescence image. This is because the device’s camera will perform an auto-exposure when the device is switched to fluorescence mode. If this auto-exposure is done under well-lit conditions (i.e., before the environment is made dark), the auto-exposure creates a false bluish colour on the image. Figure 7D–F demonstrates this effect in a diabetic foot ulcer.

Clinician are also advised to interpret fluorescent images on a colour calibrated screen (such as the imaging device itself), as computer monitors have varying levels of quality which can cause subtle features to be lost. Printed copies of fluorescence images will also be susceptible to lower colour quality.

**Figure 8:**
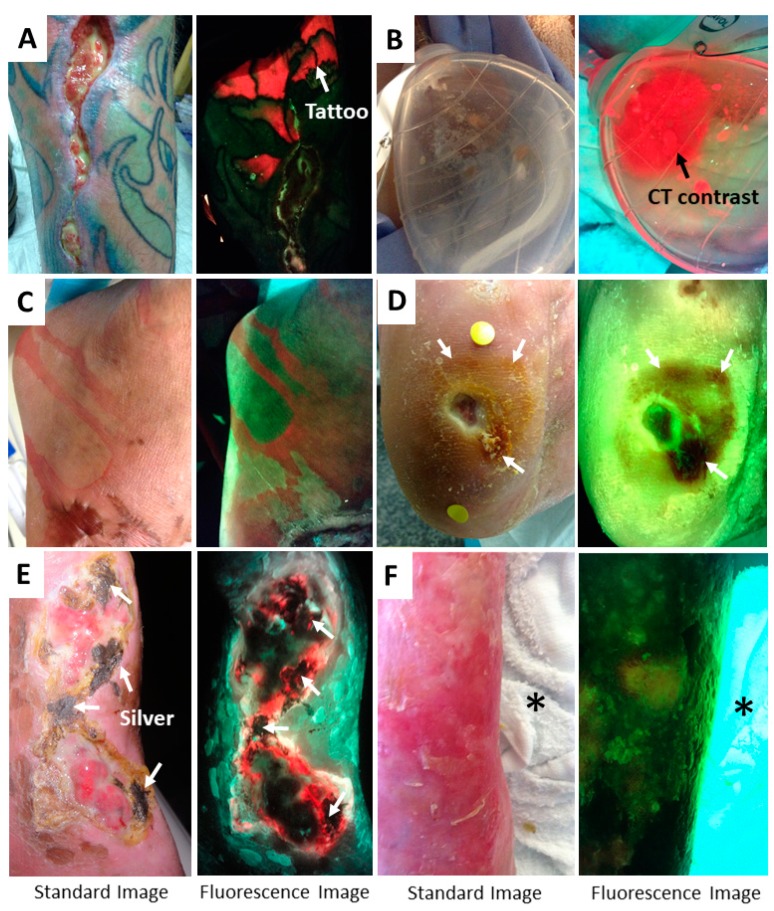
Confounding signals. (**A**) Bright red fluorescence from a tattoo. (**B**) Fluorescent red CT contrast agent observed within a Jackson Pratt wound drainage system for a patient undergoing a sinogram. (**C**) Pink chlorohexidine on a patient’s foot and ankle. This solution is tinted with pink to enable clinicians to see where the tissue has been cleaned; the tinting appears red/pink on fluorescence images. (**D**) Images of a wound after application of iodine during cleaning. Arrows denote the region of iodine, which appears reddish brown on the standard image and dark on the fluorescence image. (**E**) Venous leg ulcer with residual silver product (white arrows) on the wound. Regions with silver product on standard images result in regions of black on fluorescence images, as the violet illumination light cannot penetrate through the silver. If possible, silver products should be removed from the wound prior to imaging. (**F**) Bright fluorescence from a white bed sheet (asterisk) in the field of view. Clinicians should be aware that white items in the field of view may fluoresce.

**Figure 9:**
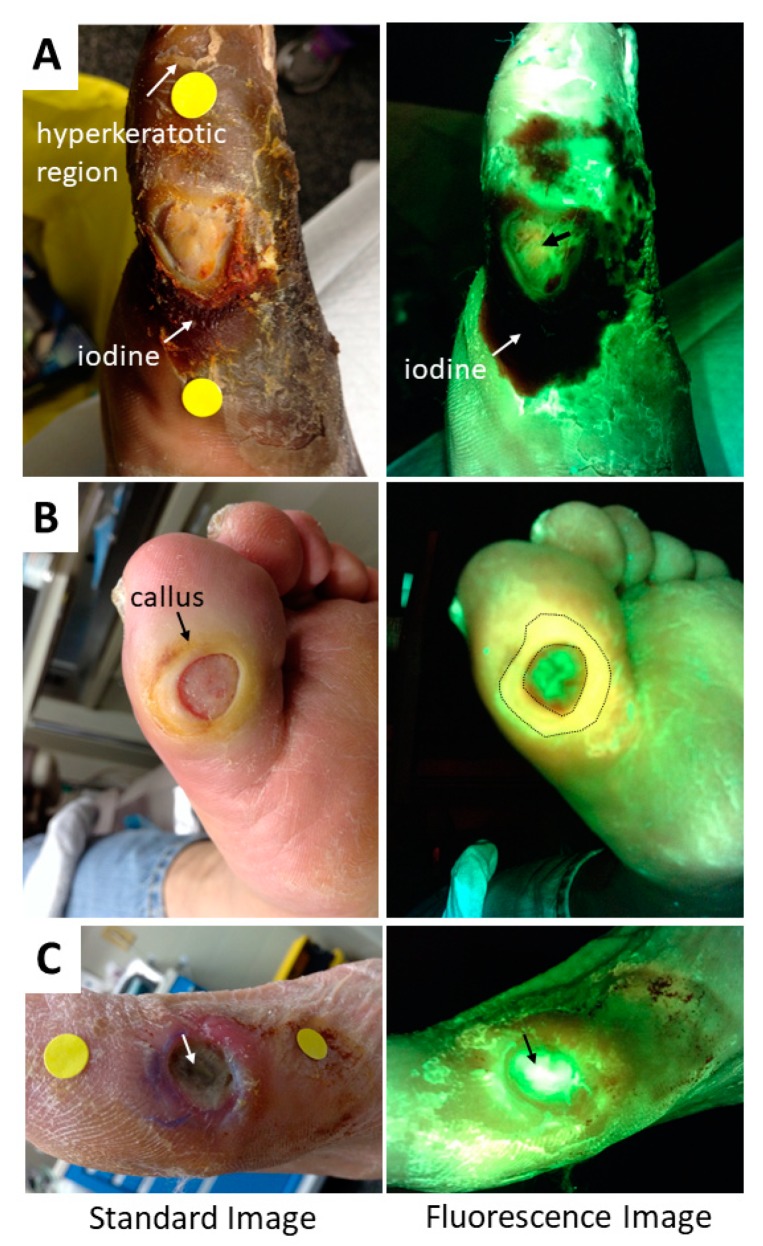
Challenging images to interpret. Standard and fluorescence images are shown for three diabetic foot ulcers (DFUs) which users may find challenging to interpret. (**A**) DFU with a region of hyperkeratosis (top of image), which fluoresces brightly (white) on fluorescence image. Iodine has been applied to the wound (reddish brown on standard image), resulting in large, dark regions on the fluorescence image. A faint blush red fluorescence was noted in the center of the wound bed, suggesting subsurface bacterial presence. A curettage sample was taken (black arrow denotes sampling location) and culture analysis found heavy growth of *Serratia marcescens*. (**B**) DFU with a heavy callus surrounding the wound. This callus tissue is expected to fluoresce bright green, however the callus region on the fluorescence image corresponded to a large region of yellow (circled). This led the clinician to suspect red bacterial fluorescence mixing with overlying green fluorescing tissue. This region was therefore sampled via curettage and cultures confirmed moderate growth of *Staphylococcus aureus*. (**C**) DFU with exposed bone (arrow) fluorescing bright white. This is shown to demonstrate how fluorescence from bone could be mistaken for *P. aeruginosa* if not compared to the standard image and assessed in conjunction with a standard clinical evaluation of the patient and their wound.

The device captures all fluorescence in the field of view, and therefore any fluorescent substances or objects in the field of view may produce a signal that is captured in the image. Most of these are obvious (e.g., fluorescent items of clothing worn by the patient). However, clinicians should be aware that fluorescent gels, fluorescent contrast agents, tattoo ink, highlighter ink around the wound, and pink chlorohexidine—which fluoresce under 405 nm excitation—will also create a fluorescent signal that may be detected by the device. Examples of these fluorescent, non-bacterial signals are shown in Figure 8. When reviewing a patient’s images, the standard and fluorescence images should be compared to one another to assist the clinician in identifying potential sources of non-bacterial signals. The device instructions for use specify that imaging should be performed after wound cleansing to remove potential contaminants and avoid this issue. Clinicians should also be aware of products that may block or absorb the fluorescence light and thus attenuate bacterial and tissue fluorescence from that region of the wound. Elemental silver, common in silver dressings, is one such product (Figure 8E). Iodine, sometimes used to cleanse wounds, will also attenuate signals and appear dark on fluorescence images (Figure 8D and Figure 9A). A summary of potential confounders is given in Table 1.

## 6. Summary

Educational resources on image interpretation are important for any new imaging technology. Fluorescence imaging with the MolecuLight i:X is a novel addition to the wound care field, as it enables real-time detection of fluorescence from bacteria at loads of clinical concern. This device also sees fluorescence characteristics from tissues and fluorescent items in the field of view. Understanding the various colours and characteristics on fluorescence images is necessary for proper assessment of whether bacterial fluorescence is present or absent. The intent of this tutorial is therefore to provide clinicians with information and relevant examples to assist in (1) their understanding of fluorescence signals and (2) their proficiency in interpreting MolecuLight i:X images. To this end, images of wounds which clinicians may find challenging to interpret are presented in Figure 9, with captions discussing the most challenging features of the images.

Numerous publications on the device have discussed its high sensitivity for bacterial detection [20,21,41,42], benefits of use during wound assessment [17,18,19,23,41], and the various wound treatments that fluorescence images can guide [19,23,24]. Ongoing and future studies with the device will evaluate the clear potential for fluorescence guided wound care to influence wound area reduction rates and wound healing.

## Figures and Tables

**Table 1:** Potential sources of fluorescence on MolecuLight i:X images.

	Bacteria	Tissue Components	Confounders
Red	Porphyrin producing bacteria ^1^	–	Ambient light contamination Tinted cleansing solutions (e.g., pink chlorohexidine) Tattoo with red dye
Cyan	*Pseudomonas aeruginosa*	–	White cotton (including bed linens, surgical scrubs, clothing) Paper products (e.g., measuring ruler) Blue absorbent pad Tattoo with blue dye
White	*Pseudomonas aeruginosa*	Tendon, bone, flaky skin, nails	White cotton (including bed linens, surgical scrubs, clothing) Paper products (e.g., measuring ruler) Dressings (e.g., gauze, foam bandages)
Dark (black/maroon)	–	Hemoglobin (blood or highly vascular tissue) Necrotic tissue	Iodine Silver dressing Gentian violet
Green	–	Skin, slough	Collagenase ointment

^1^ Includes gram-negative and gram-positive species, aerobes and anaerobes (e.g., *Staphylococcus*, *E. coli*, *Klebsiella*, *Proteus*, *Enterobacter*, *Acinetobacter, Aeromonas, Bacteroides,* others) [46].
